# Repurposing *Astragalus* Polysaccharide PG2 for Inhibiting ACE2 and SARS-CoV-2 Spike Syncytial Formation and Anti-Inflammatory Effects

**DOI:** 10.3390/v15030641

**Published:** 2023-02-27

**Authors:** Chia-Yin Lee, Anh Thuc Nguyen, Ly Hien Doan, Li-Wei Chu, Chih-Hung Chang, Hui-Kang Liu, I-Lin Lee, Teng-Hsu Wang, Jin-Mei Lai, Shih-Ming Tsao, Hsiu-Jung Liao, Yueh-Hsin Ping, Chi-Ying F. Huang

**Affiliations:** 1 Taiwan National Graduate Program in Molecular Medicine, National Yang Ming Chiao Tung University and Academia Sinica, Taipei 112304, Taiwan; 2 Institute of Biopharmaceutical Sciences, College of Pharmaceutical Sciences, National Yang Ming Chiao Tung University, Taipei 112304, Taiwan; 3 Department and Institute of Pharmacology, College of Medicine, National Yang Ming Chiao Tung University, Taipei 112304, Taiwan; 4 Department of Orthopedic Surgery, Far Eastern Memorial Hospital, New Taipei City 220216, Taiwan; 5 Division of Basic Chinese Medicine, National Research Institute of Chinese Medicine (NRICM), Ministry of Health and Welfare, Taipei 112304, Taiwan; 6 PhytoHeath Corporation, Taipei 105403, Taiwan; 7 Department of Life Science, College of Science and Engineering, Fu Jen Catholic University, New Taipei City 242062, Taiwan; 8 Division of Pulmonary Medicine, School of Medicine, Chung Shan Medical University Hospital, Chung Shan Medical University, Taichung 402306, Taiwan; 9 Department of Medical Research, Far Eastern Memorial Hospital, New Taipei City 220216, Taiwan

**Keywords:** COVID-19, microRNAs, macrophages, cytokine storm, viral entry

## Abstract

The outbreak of coronavirus disease 2019 (COVID-19) caused by severe acute respiratory syndrome coronavirus 2 (SARS-CoV-2) poses a serious threat to global public health. In an effort to develop novel anti-coronavirus therapeutics and achieve prophylactics, we used gene set enrichment analysis (GSEA) for drug screening and identified that *Astragalus* polysaccharide (PG2), a mixture of polysaccharides purified from *Astragalus membranaceus*, could effectively reverse COVID-19 signature genes. Further biological assays revealed that PG2 could prevent the fusion of BHK21-expressing wild-type (WT) viral spike (S) protein and Calu-3-expressing ACE2. Additionally, it specifically prevents the binding of recombinant viral S of WT, alpha, and beta strains to ACE2 receptor in our non-cell-based system. In addition, PG2 enhances *let-7a*, *miR-146a*, and *miR-148b* expression levels in the lung epithelial cells. These findings speculate that PG2 has the potential to reduce viral replication in lung and cytokine storm via these PG2-induced miRNAs. Furthermore, macrophage activation is one of the primary issues leading to the complicated condition of COVID-19 patients, and our results revealed that PG2 could regulate the activation of macrophages by promoting the polarization of THP-1-derived macrophages into an anti-inflammatory phenotype. In this study, PG2 stimulated M2 macrophage activation and increased the expression levels of anti-inflammatory cytokines IL-10 and IL-1RN. Additionally, PG2 was recently used to treat patients with severe COVID-19 symptoms by reducing the neutrophil-to-lymphocyte ratio (NLR). Therefore, our data suggest that PG2, a repurposed drug, possesses the potential to prevent WT SARS-CoV-2 S-mediated syncytia formation with the host cells; it also inhibits the binding of S proteins of WT, alpha, and beta strains to the recombinant ACE2 and halts severe COVID-19 development by regulating the polarization of macrophages to M2 cells.

## 1. Introduction

Severe acute respiratory syndrome coronavirus 2 (SARS-CoV-2), a positive-strand-enveloped RNA virus identified during the pneumonia outbreak in December 2019, causes a human disease called coronavirus disease 2019 (COVID-19) [1]. During viral replication, the spike (S) protein cleaves into two subunits, S1 and S2. S1 is responsible for binding to host cell angiotensin-converting enzyme 2 (ACE2) receptors, and S2 is primarily involved in regulating viral fusion and entry into the host cell by forming a six-helical bundle via the two-heptad repeat domain [2]. Viral enzymes, proteins, and cytokines involved in virus entry, replication, and host macrophage overreaction are potential drug targets for the development of therapeutic options for SARS-CoV-2 [3].

Patients initially infected with SARS-CoV-2 have symptoms similar to those of the flu. Rapid replication of the viral genome leads to continuous activation of the immune system. Macrophages are multifaceted within the alveolar space, and their distinct functions depend highly on their polarization, generally characterized as M1 or M2 macrophages. Based on distinct gene expression and functional profiles, M1 macrophages are proinflammatory macrophages that are crucial against pathogens, whereas M2 macrophages inhibit inflammation and modulate the repair of damaged tissues [4,5]. Notably, M1 macrophages dampen the growth and enhance the apoptosis of lung cells. Conversely, inhibition of viral entry using an ACE2-blocking antibody substantially enhances the activity of M2 macrophages [6]. Thus, we propose that increased M2 macrophage polarization improves the inflammatory lung microenvironment. Due to permanent damage to alveolar and endothelial cells and alveolar–capillary barrier interruption, patients commonly present with dyspnea and persistent symptoms of viral pneumonia, including decreased oxygen saturation and lymphocytosis, together with ground-glass images and alveolar exudates with intralobular involvement on lung imaging. As a result, these symptoms rapidly progress to severe acute lung injury—called acute respiratory distress syndrome (ARDS) [7,8]—and septic shock, eventually leading to multiple organ failure. Due to an excessive immune response, surviving patients suffer from long-term symptoms, including fatigue, dyspnea, and cognitive dysfunction and generally have an impact on everyday functioning [9]. Currently, there is no fully proven treatment for COVID-19 patients with excessive inflammation. Some anti-inflammatory and antifibrotic drugs, such as pirfenidone and nintedanib, are considered to reduce ARDS-induced lung damage. However, neither has been studied in the case of acute exacerbations nor ARDS due to the possible consequence of liver toxicity and increased patient’s risk of bleeding [10,11,12].

PG2^®^ lyophilized injection (PhytoHealth Corporation, Taipei, Taiwan) is an injectable medicinal product containing an active fraction of *Astragalus* polysaccharides which is extracted, isolated, and purified from the roots of *Astragalus membranaceus* (Huangqi in Chinese). The roots of *Astragalus membranaceus*, also known as *Astragali radix*, have long been used to treat “*qi* deficiency (lack of energy)” in traditional Chinese medicine (TCM) [13,14]. It has been clinically proven to ease fatigue among advanced cancer patients and has been approved by the Taiwan Food and Drug Administration (TFDA) as a prescription drug for relieving cancer-related fatigue (CRF).

As included in the approved label of PG2, it can stimulate medullary hematopoiesis and enhance immune function [15]. It was reported that PG2 can increase the activity of natural killer cells (NK cells) and production of IL-2 as well as enhance resistance in normal mice by regulating the immune system and prolonging survival under the dual insults from X-ray radiation at the median lethal dose and invasion by cytomegalovirus (CMV) [15]. Current clinical research has reported that PG2 could decrease the neutrophil-to-lymphocyte ratio (NLR), which is a chronic inflammation marker and a prognostic indicator of worse clinical outcomes in several cancer types [16]. NLR on admission is also considered a predictor of the severity and mortality of COVID-19 patients in the latest reports [17,18]. Wang et al. recently reviewed the successful management of COVID-19 cases at Chung-shan Medical University Hospital, Taichung, Taiwan, including four patients receiving standard care who had high NLRs and marked lymphopenia and were treated with PG2 during hospitalization [19]. The decrease in NLR in these four patients was faster than that in other patients who did not receive PG2, and all four cases successfully recovered from severe COVID-19 symptoms [19]. The current report sheds light on the positive effects of *Astragalus* polysaccharides against life-threatening outcomes of the disease in COVID-19 patients.

Based on our previous research on in silico analysis, *Astragalus* polysaccharides were identified to be a promising treatment for COVID-19 [20]. Gene set enrichment analysis (GSEA) of the PG2 treatment transcriptional profile showed that PG2 can reverse COVID-19 signatures (MSigDB database) as well as ARDS (GSE76293), thus providing the rationale for PG2 as a potential therapy against SARS-CoV-2 infection. In addition, biological experiments also indicated that *Astragalus* polysaccharides inhibited the binding of SARS-CoV-2 S protein to ACE2 and blocked the fusion ability of the S protein. Furthermore, in lung epithelial cells, PG2 treatment can increase the expression levels of *let-7a*, *miR-146a*, and *miR-148b*, whose functions have been shown to target the viral genome to prevent viral replication and regulate the Toll-like receptor (TLR) signaling pathway to prevent overactivation of macrophages and cytokine storm [7,21,22]. Our study elucidates PG2 mechanisms that support its benefits for future usage; PG2 can elevate miRNAs having antiviral potential and anti-inflammatory ability of macrophages, thereby driving M2 macrophage polarization.

## 2. Materials and Methods

### 2.1. PG2 Injection

PG2 Injection was provided by PhytoHealth Corporation (Taipei, Taiwan) through a series of proprietary processes—including water extraction of *Astragalus membraneous* (AM) root, ethanol precipitation, condensation, filtration, purification, and spray drying—to obtain highly purified (>90%) polysaccharides with average molecular weights ranging from 20,000 to 60,000 Da. The major polysaccharides in this product were α-1,4-linked glucans, with a certain degree of branching at six positions on the backbone residues. Other components of this product included arabinogalactans, rhamnogalacturonans, and arabinogalactan proteins. The composition of PG2 and its determination methods have been previously described in [23].

### 2.2. L1000 Gene Expression Profiling 

M2 cells were treated with PG2 (32 mg/mL) for 6 h, and gene expression profiling was performed using L1000 expression profiling by Genometry, Inc., Cambridge, MA, USA. The L1000 profiling protocol includes the capture of mRNA transcripts from whole-cell lysates by oligo-dT followed by the generation of cDNAs via reverse transcription and amplification using polymerase chain reaction (PCR). To determine the expression levels of 978 landmark genes, the PCR amplicon was hybridized to barcoded Luminex beads, and specific probes were annealed to the cDNA.

### 2.3. Gene Set Enrichment Analysis (GSEA) 

For each gene set, GSEA determined the enrichment score and subsequently normalized the score according to its size. The score can be positive or negative, which demonstrates that a gene set is enriched at the top or bottom of the list of ranked genes, respectively, based on expression levels. The C2 gene set collection included in the MSigDB v.7.4. database was utilized in the GSEA in this study. The GSE76293 microarray for ARDS signature was retrieved from the Gene Expression Omnibus (GEO), which was pre-processed using the limma package. GSEA was performed using 1000 permutations with a gene set permutation type.

### 2.4. Cell Culture 

Baby hamster kidney (BHK)-21 cells, a fibroblast cell line derived from baby hamster kidneys (ATCC, cat. no. CCL-10), were cultured in Dulbecco’s Modified Eagle Medium (DMEM, Gibco, cat. no. 12100046) and supplemented with 10% FBS and 1 × penicillin/streptomycin solution. BHK-21 was incubated at 37 °C in a 5% CO_2_ atmosphere and trypsinized every 2 days. After seeding for 24 h, cells were used for plasmid transfection and cytotoxicity assay. Calu-3 cells, a human epithelial lung cell line derived from a patient with lung adenocarcinoma (ATCC, cat. no. HTB-55), were cultured in Dulbecco’s Modified Eagle Medium (DMEM, Gibco, cat. no. 12100046) and supplemented with 10% FBS and 1 × penicillin/ streptomycin solution. Calu-3 was incubated at 37 °C in a 5% CO_2_ atmosphere and trypsinized every 3–4 days. After seeding for 48 h, cells were used for cell–cell fusion assay, cytotoxicity assay, and western blotting assay. BEAS-2B, a human normal bronchial epithelial cell line (ATCC, cat. no. CRL-9609), was cultured in RPMI medium supplemented with 10% FBS (Invitrogen, ThermoFisher, Waltham, MA, USA), 1% PSA, 1% nonessential amino acid, and 2 mM L-glutamate (Invitrogen). Cells were maintained at 37 °C with 5% CO_2_ in a cell incubator and passaged every 3–4 days. For the experiment, the cell line was used at an early passage (before passage 6). The human monocytes THP-1 cell line (BCRC, cat. no. 60430) used in this study was obtained from the Bioresource Collection and Research Center (BCRC) and was cultured in RPMI 1640 medium with high glucose (Gibco, cat. no. A1049101). The RPMI medium was supplemented with 10% FBS (Gibco, cat. no. A2720803), 100 IU/mL penicillin, and 0.1 mg/mL streptomycin. THP-1 monocytes were differentiated into macrophages by 24 h incubation with phorbol 12-myristate 13-acetate (PMA) at 50 ng/mL followed by 24 h incubation in RPMI medium for ELISA, qRT-PCR, and western blotting assay.

### 2.5. Cell–Cell Fusion Assay 

The assay was performed as previously described [24]. Briefly, on a six-well plate, donor BHK-21 cells were seeded at 4 × 10^5^ cells/well and transfected with both EGFP and S plasmids (Wuhan strain) at a ratio of 1:5 using Lipofectamine™ 2000 Transfection Reagent (Invitrogen, cat. no. 11668019). After 24 h, 1 mL of enzyme-free PBS-based cell dissociation buffer (Gibco, cat. no. 13151014) was added to each well, and the cells were allowed to resuspend in serum-free DMEM (Gibco, ThermoFisher, Waltham, MA, USA). The cells were then cocultured with human lung cancer Calu-3 cells seeded in a single layer to induce cell–cell interactions in the presence or absence of PG2 (0.2, 0.67, or 2 mg/mL). The cocultured cells were then incubated at 4 °C for 1 h. The unbound cells were then removed using PBS, and the growth medium was replaced. Initial images of EGFP-positive cells in five random areas were used to evaluate the binding efficiency. An inverted fluorescence microscope (Olympus IX70) was used to capture the images. Furthermore, the corresponding doses of PG2 were added and incubated for 4 h at 37 °C. Images of five fields of EGFP-positive cells were randomly taken as previously described, indicating fusion efficiency. The quantification of binding efficiency and syncytial cell formation was performed as previously described [24].

### 2.6. Trimeric S Protein Assay 

Enzyme-linked immunosorbent assay (ELISA) was performed to examine the ability of PG2 to intervene in the ability of wild-type trimeric SARS-CoV-2 S protein (Wuhan strain) or variants (α, β, γ, δ) to bind to biotinylated human ACE2 recombinant protein. The experimental procedures were performed as previously described [24]. Briefly, each well of a 96-well plate was coated overnight at 4 °C with 500 ng/mL of S protein (Cat. no. GTX135972-pro; GeneTex, Irvine, CA, USA) diluted in coating buffer containing 15 mM sodium carbonate and 35 mM sodium hydrogen carbonate at pH 9.6. The next day, after washing three times with washing buffer (PBS with 0.05% (*v*/*v*), Tween-20 (pH 7.4)), 300 μL of blocking buffer prepared from washing buffer supplemented with 0.5% (*w*/*v*) bovine serum albumin (BSA) for 1.5 h at 37 °C was added. Different doses of PG2 (1, 2, 4, 8 mg/mL) or 10 μg/mL of inhibitor (GeneTex, cat. no. GTX635791) were then replaced and incubated for 1 h at 37 °C. Next, the plate was incubated at 37 °C for 1 h with 125 ng/mL of biotinylated human ACE2 protein (Cat. no. AC2-H82E6-25ug; ACROBiosystems, OX, London, UK). Next, 100 ng/mL/well of Streptavidin-HRP conjugates (GeneTex, cat. no. GTX635791) was added and incubated for another 1 h at 37 °C. Finally, the washed plate was incubated with 200 μL of TMB substrate per well for an additional 1 h at 37 °C in the dark. A stop solution (50 µL) was used to block the reaction. The absorbance was measured at 450 nm using a microplate reader (Cytation 5, BioTek, Winooski, VT, USA).

### 2.7. qRT-PCR Analysis

BEAS2B cells were used to screen for biological agents that affect respiratory tract infection mechanisms. To determine whether the candidate drugs can induce *let-7a*, *miR-148*, and *miR-146* expression levels, 1 × 10^6^ cells were seeded in a 10-cm dish for at least 16 h before 24 h of drug treatment. Total RNA from the cells was extracted using the RNA Extraction Kit (Cat. no. DR100; Geneaid, New Taipei City, Taiwan). TRIzol reagent (Invitrogen) was used to lyse cells, allowing DNA to bind to the genomic DNA mini spin column. The flow-through containing RNA was then transferred to the RNA mini spin column for RNA binding. Contaminants were removed using a wash buffer, and the purified total RNA was eluted in RNase-free water. This process was followed according to the manufacturer’s instructions. Additionally, 1 μg of total RNA was used for reverse transcription using the SuperScript III First-Strand Synthesis Kit (Invitrogen) and oligo-dT priming as per the manufacturer’s instructions. For qPCR, cDNA was amplified using SYBR green PCR master mix (Applied Biosystems, ThermoFisher, Waltham, MA, USA). The real-time PCR primers used in the assay were forward sequences specific for *has-let-7a-5p* (5′-GCCTGAGGTAGTAGGTTGTATAGTTA-3′), *hsa-miR148b-5p* (5′-AAGUUCUGUUAUACACUCAGGC-3′), *hsa-miR-146a* (5′-UGAGAACUGAAUUCCAUGGGUU-3′), and U54 (homo) (5′-GGTACCTATTGTGTTGAGTAACGGTGA-3′). *U54* was used as an internal control.

### 2.8. Western Blotting 

Cells were exposed to different treatments at different time points as indicated in the Section 3 and figure legends; later, they were harvested and lysed on ice with RIPA lysis buffer, followed by centrifugation at 12,000× *g* for 10 min. The lysate was subjected to sodium dodecyl sulfate-polyacrylamide gel electrophoresis, and the proteins were electrotransferred onto polyvinylidene fluoride membrane. The membrane was then blocked with 5% non-fat dry milk in Tris-buffered saline at room temperature for 1 h and incubated with the primary antibody overnight, followed by secondary antibody incubation for 1 h. Immunoreactive bands were visualized using enhanced chemiluminescence (ECL) (Thermo cat. no. 34580). Images were captured using a Luminescence Imaging system (LAS 4000™M, Fuji Photo Film Co., Ltd., Minato, Tokyo, Japan). The densities of the bands were measured using Image J software. The ratio of proteins to the internal control (GAPDH or α-tublin) were calculated. The relative expression levels of proteins were normalized to the control group, which was assigned a value of 1. The following antibodies were used: rabbit anti-CD163 monoclonal antibody (1:1000, cat. no. ab182422; Abcam Inc., Toronto, ON, Canada); rabbit anti-CD86 monoclonal antibody (1:1000, Abcam, cat. no. ab239075); rabbit anti-GAPDH monoclonal antibody (1:10,000, GeneTex, cat. no. GTX100118); Peroxidase AffiniPure Goat Anti-Rabbit IgG (1:5000, Jackson Lab, cat. no. 111-035-045); rabbit anti-ACE2 monoclonal antibody (1:3000, GeneTex, cat. no. GTX01160); rabbit anti-TMPRSS2 polyclonal antibody (1:2000, GeneTex, cat. no. GTX64544); and rabbit anti-α-tubulin polyclonal antibody (1:3000, GeneTex, cat. no. GTX112141).

### 2.9. Induction of Human M2 Macrophages and FACS Analysis 

Following a literature protocol [25], human macrophage preparation was conducted by freshly isolating peripheral mononuclear cells from the blood of healthy donors following standard density gradient centrifugation with Ficoll-Paque. CD14^+^ cells from peripheral mononuclear cells were purified via high-gradient magnetic sorting. CD14^+^ monocytes were cultured for six days in a complete RPMI-1640 medium supplemented with 10 ng/mL human macrophage colony-stimulating factor (M-CSF) for the induction of macrophages. CD14^+^ monocytes were differentiated into an M2-like phenotype: M-CSF for seven days, followed by 20 ng/mL IL-4 for an additional day. For FACS analysis, human M2 macrophages were stained with monoclonal mouse anti-human CD163 (Cat. no. 155306; BioLegend, San Diego, CA, USA) and CD206 (mannose receptor, MR) antibodies (BioLegend, cat. no. 321152). The cells were suspended in PBS at a density of 1 × 10^5^ cells/mL. A 5% BSA buffer was used to block nonspecific antigens. The cells were analyzed using a CytoFLEX flow cytometer (Beckman Coulter, Brea, CA, USA). Nonspecific mouse immunoglobulin was used as the isotype control. For measuring the production of IL-10 and IL-1RN, ELISA was used. Concentrations of IL-10 (BioLegend, cat. no. 430604) and IL-1RN (Cat. no. DRA00B; R&D Systems, Minneapolis, MN, USA) levels in the supernatants of human M2-like macrophages were measured using commercially available ELISA kits (Human IL-1RN, R&D, Cat. no. DY280; Human IL-10, R&D, Cat. no. DY217B). The ELISA was performed according to the manufacturer’s instructions.

### 2.10. Statistics 

Data are presented as the mean ± standard error of the mean. Statistical calculations were performed using the GraphPad Prism software (GraphPad, San Diego, CA, USA). One-way analysis ANOVA with Dunnett’s test was used for multiple comparisons. Single-parameter comparisons were performed using unpaired Student’s *t*-tests. *p*-values of less than 0.05, 0.01, and 0.001 were considered significant.

## 3. Results

### 3.1. GSEA Reveals the Effect of PG2 on Reversing COVID-19 Signatures

**Figure 1:**
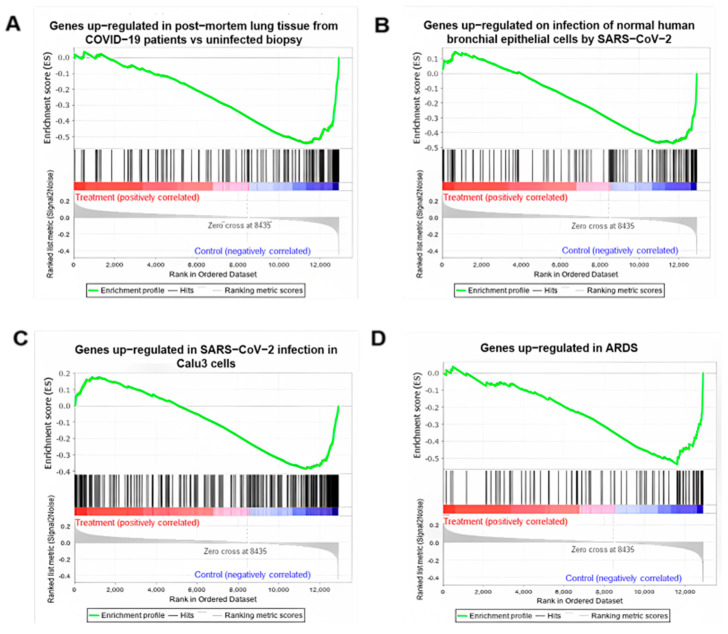
GSEA reveals reversing effect of PG2 on COVID-19 signature. PG2 treatment GSEA enrichment plot of gene sets on (**A**) upregulated genes in post-mortem lung tissue from COVID-19 patients vs. uninfected biopsy; (**B**) upregulated genes on infection following normal human bronchial epithelial cells by SARS-CoV-2; (**C**) upregulated genes in SARS-CoV-2 infection in Calu-3 cells; and (**D**) genes upregulated in ARDS (GSE76293).

Gene expression profiles of M2 macrophages treated with PG2 were analyzed via GSEA to identify potential novel indications, as well as the underlying mechanism. Interestingly, GSEA suggested that treatment with PG2 could reverse the SARS-CoV-2 infection signatures; the genes increased/decreased in the disease conditions can be decreased/increased after drug treatment. The COVID-19 signature was described by Blanco-Melo et al. [26] as genes being up/downregulated by SARS-CoV2 infection compared to other respiratory viruses, indicating unique signatures of COVID-19. From the transcriptional responses to PG2 treatment, we observed that the SARS-CoV-2 signature in Calu-3 and bronchial epithelial cells was reduced, in which genes upregulated by SARS-CoV-2 infection were downregulated (NES < 0). Moreover, strongly enhanced genes in postmortem lung samples from COVID-19-positive patients were also decreased by PG2 treatment (NES = −2.58) (Table 1, Figure 1A–C). In contrast, the GSEA results also indicated an anti-SARS-CoV-2 effect of PG2 via a reduction in the gene set of the COVID-19 adverse outcome pathway (WikiPathways—WP4891), maturation of the SARS-CoV-2 S protein (Reactome—R-HSA-9694548), translation of SARS-CoV-2 structural proteins (Reactome—R-HSA-9694635), and SARS-CoV-2 infection (Reactome—R-HSA-9694516) (Table 1, Supplementary Figure S1A–D). The effect of PG2 on ARDS was also investigated using the ARDS signature obtained by significantly upregulated genes in blood polymorphonuclear neutrophils (PMNs) from patients with ARDS (GSE76293). A negative NES indicated that PG2 was able to reduce the number of genes upregulated in ARDS patients (Table 1, Figure 1D).

Given that ferroptosis has been elucidated in SARS-CoV-2-induced multi-organ damage [27], PG2 might also reduce ferroptosis to cure COVID-19 as the transcriptional profile of PG2 treatment showed a significant decrease in gene sets related to ferroptosis (WikiPathways—WP4313) and iron uptake and transport (Reactome—R-HSA-917937) (Table 1, Supplementary Figure S1E,F).

### 3.2. Suppression of SARS-CoV-2 S Protein-Mediated Syncytium Formation by PG2

**Figure 2:**
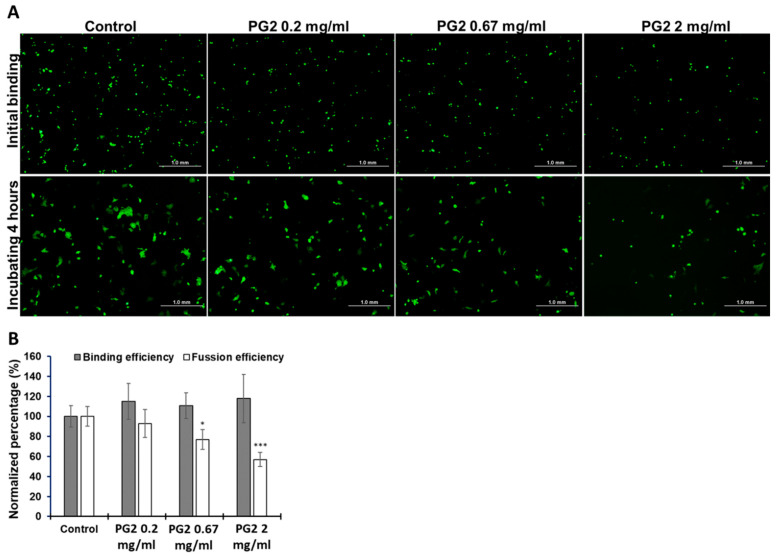
PG2 inhibits WT SARS-CoV-2 S-mediated syncytia formation. (**A**) EGFP and wild-type S protein coexpressed BHK21 cells were added into Calu-3 cells and incubated at 4 °C for 1 h for S-ACE2 binding. After 4 h of incubation, large fluorescent multinucleate cells were formed in the control group, indicating S-mediated syncytium formation. The histogram depicts the binding and fusion efficiency in each group (*n* = 3). The binding efficiency of the SARS-CoV-2 S to ACE2 (gray bars) and the formation of syncytium indicating fusion efficiency (white bars) was quantified in (**B**). *p < 0.05, *** *p* < 0.001. Scale bars, 1.0 mm.

Receptor-dependent syncytial formation promoted by the SARS-CoV-2 S protein on the cell membrane is a crucial step in the cellular invasion of SARS-CoV-2 [28,29,30]. Indeed, clinical evidence of infected syncytial pneumocytes in COVID-19 patients who died implies a high correlation between pneumocyte syncytia and severe COVID-19 pathogenesis [31,32]. Hence, we investigated the binding efficiency of EGFP-S-positive BHK-21 cells and hACE2-receptor-expressing Calu-3 cells treated with PG2 in a cell–cell fusion assay. The attachment between BHK-21 cells and Calu-3 cells represents the binding of the SARS-CoV-2 S protein to the ACE2 receptor. Syncytial formation caused by the fusion of BHK-21 and Calu-3 cells was quantified to evaluate the PG2 inhibitory effect. As shown in Figure 2, there was no significant difference in the number of EGFP-positive cells between the three PG2-treated groups and the control group (Figure 2A, upper panel; Figure 2B, gray bars). After an additional 4 h of incubation, multinucleated giant cells with expanded EGFP signals were detected in the control treatment, suggesting the formation of a S-mediated syncytium. Compared to the control group, more than 20% and 40% of the area of EGFP-positive syncytium was reduced in the presence of 0.67 and 2 mg/mL PG2 treatment, respectively (Figure 2A, bottom panel; Figure 2B, white bars). However, the unchanged expression levels of ACE2 and TMPRSS2 after 5 h of PG2 treatment were validated by western blot (Supplementary Figure S2). These results indicate that PG2 could suppress the formation of WT SARS-CoV-2 S protein-mediated syncytium in a dose-dependent manner between BHK-21 and Calu-3 cells in an in vitro system. The current results suggest that PG2 could be effective against SARS-CoV-2 by inhibiting membrane fusion. However, this needs to be validated by inducing SARS-CoV-2 infection and utilizing PG2 to study its efficiency.

### 3.3. The Inhibitory Effect of PG2 on the Binding of SARS-CoV-2 S Protein with ACE2 Is Variant Specific

**Figure 3:**
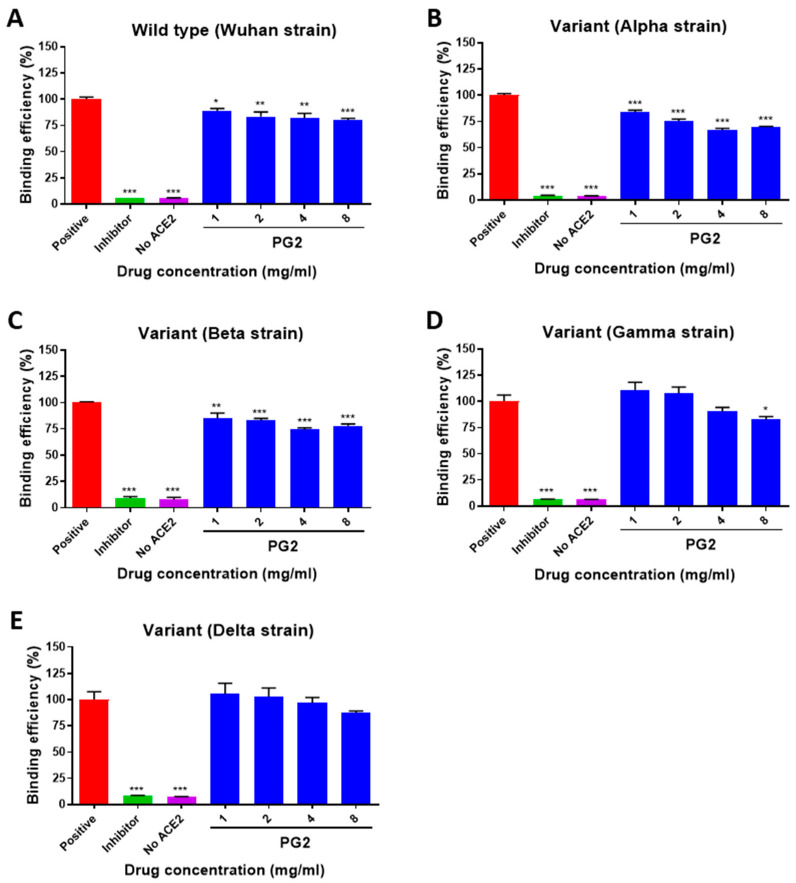
Variant specificity of PG2 against trimeric S protein binding to ACE2. Trimeric S proteins derived from (**A**) wild type/Wuhan strain, (**B**) variant alpha strain, (**C**) variant beta strain, (**D**) variant gamma strain, and (**E**) variant delta strain were used for ELISA-based S protein and hACE2 binding assays. Positive condition represents full binding activity of trimeric S protein on hACE2. The inhibitor is the RBD antibody (10 μg/mL). Data are represented as mean ± SEM (*n* = 4). * *p* < 0.05, ** *p* < 0.01, and *** *p* < 0.001 when compared to the binding efficiency of the positive group.

We compared the potential inhibitory effects of PG2 on the current circulating variants by employing an ELISA-based trimeric S protein binding assay. As shown in Figure 3, the presence of PG2 at concentrations between 1 and 8 mg/mL marginally reduced the binding efficiency of the trimeric S protein from the wild-type, alpha, and beta variants (Figure 3A–C). Among these three types, PG2 appeared to be more effective when confronted with S proteins from the alpha and beta strains. In contrast, PG2 had only a modest effect on the gamma variant (Figure 3D) and did not affect the delta variant (Figure 3E).

### 3.4. PG2 Elevates Let-7a, miR-148b, and miR-146a Expression Levels

**Figure 4:**
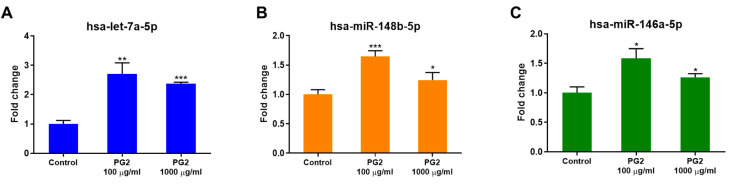
Induction of *let-7a*, *miR-148b*, and *miR-146a* levels by PG2. *Let-7a* (**A**), *miR-148b* (**B**), and *miR-146a* (**C**) expressions were measured by qRT-PCR after 24 h treated with PG2 100 or 1000 µg/mL. Statistical analysis was performed using a one-way ANOVA. *, **, or ***: significantly different from the corresponding control respectively with *p* < 0.05, 0.01, or 0.001.

Previous studies demonstrated that condensed extracts, APS and APS-L, from *Astragalus* polysaccharides could effectively block inflammation and viral replication by increasing the levels of *let-7a*, *miR-148b*, and *miR-146a* [20]. Thus, we examined the efficacy of PG2, the highly purified *Astragalus* polysaccharides, on inducing these targeted miRNAs by measuring their expression levels in BEAS2B cells after 24 h of PG2 treatment using qRT-PCR. The results indicated that PG2 was efficient in enhancing the expression of all three targeted miRNAs at both doses, but the effect of the low dose was slightly more prominent than that of the high dose. In this part, PG2 100 and 1000 µg/mL significantly augmented *let-7a*, *miR-148b*, and *miR-146a* expression 2.7- and 2.4-fold, 1.6- and 1.2-fold, and 1.6- and 1.3-fold (Figure 4A–C), respectively. These results suggested that PG2 can effectively enhance *let-7a*, *miR-148b*, and *miR-146a* expression levels.

### 3.5. PG2 Differentiates THP-1-Derived Macrophages into M2-like Phenotype

**Figure 5:**
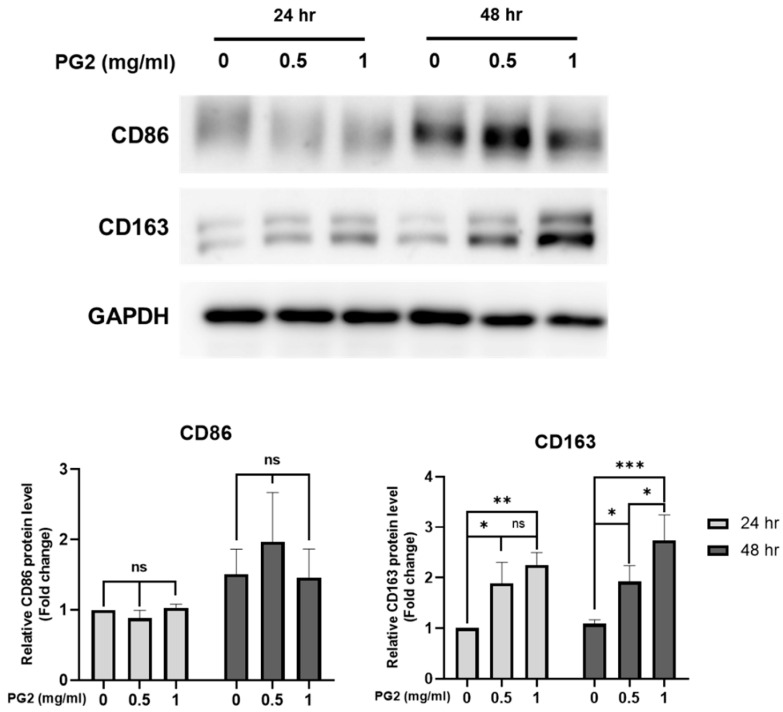
PG2 increases the expression levels of CD163 in THP-1-derived macrophages. THP-1-derived macrophages were treated with different PG2 concentrations for 24 or 48 h. Whole-cell lysates were prepared and subjected to western blotting. GAPDH was used as an internal control. The vehicle group (M0, 24 h) was used as 1 for a fold change. Data are presented as mean ± S.D. (*n* = 3). * *p* < 0.05, ** *p* < 0.01, *** *p* < 0.001, and ns means no significant difference.

To investigate the polarization effect in macrophages of PG2, THP-1 monocytes were incubated with PMA (50 ng/mL) for 24 h, followed by incubation with fresh medium to differentiate M0-type macrophages. M0-type macrophages were treated with different PG2 doses for 24 and 48 h. CD163, a scavenger receptor, is widely used as a marker for M2 macrophage polarization and inflammation [33]. According to the western blotting results, the expression levels of the CD163 significantly increased in a dose- and time-dependent manner post-incubation with PG2. These results indicated that PG2 could polarize macrophages toward the M2 type (anti-inflammatory type) (Figure 5).

### 3.6. PG2 Stimulates Human Monocyte to M2 Macrophage Transition

M2 macrophages have anti-inflammatory functions and alleviate cytokine storms in severe COVID-19 [6]. To further investigate whether PG2 might increase M2 polarization, human-monocyte-derived macrophages were treated with either PG2 or IL-4. The interleukin 4 receptor (IL-4R) can bind to IL-4 and induce anti-inflammatory immune activity. IL-4 was reported to activate M2 macrophages that express CD163 and CD206 markers [34] and anti-inflammatory cytokines, such as IL-10 and IL-1R antagonist (IL-1RN) [35]. M2 macrophages have anti-inflammatory functions and alleviate cytokine storms in severe COVID-19 [6].

**Figure 6:**
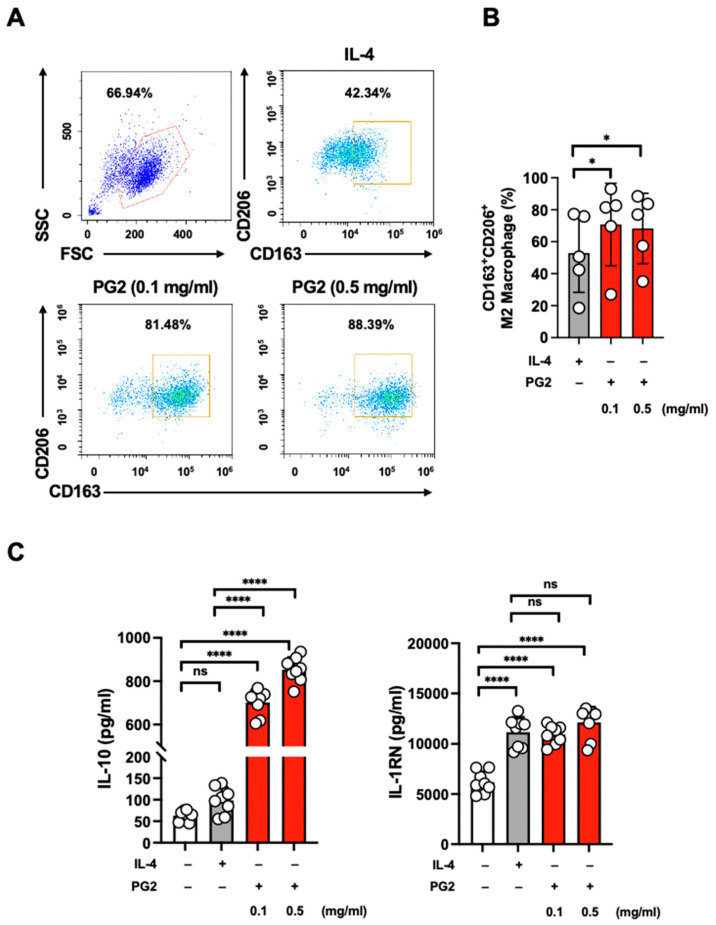
PG2 stimulates human M2 macrophage differentiation. (**A**) Flow cytometric data depicting the percentage of CD163^+^CD206^+^ expressing populations after PG2 treatment on day 7. (**B**) Quantification of the CD163^+^CD206^+^ populations by PG2. (**C**) IL-10 and IL-1RN production were stimulated by PG2 using ELISA. One-way ANOVA was performed. * *p* < 0.05, **** *p* < 0.0001 for treatments versus M2 skewing or indicated groups.

To determine the effectiveness of PG2 in promoting anti-inflammatory capacity, we investigated whether PG2 could stimulate M2 macrophage polarization. To analyze whether PG2 could modify macrophage differentiation, PG2 cells were treated with CD14-enriched cells and evaluated for the induction of macrophage-associated cell surface antigens described above. M2 macrophage differentiation was demonstrated by the upregulation of CD163 and CD206 (Figure 6A,B). Interestingly, CD14-enriched cells exposed to PG2 showed increased protein expression of CD163 and CD206 without IL-4 stimulation. As anticipated, the percentage of CD163^+^CD206^+^-expressing M2 macrophages treated with PG2 was significantly increased, as confirmed by flow cytometry. Furthermore, the anti-inflammatory cytokines IL-10 and IL-1RN were increased during M2 macrophage polarization. Our results showed that PG2 significantly increased IL-10 and IL-1RN levels (Figure 6C). These results indicated the anti-inflammatory capacity of PG2 by stimulating M2 macrophage differentiation and anti-inflammatory cytokine secretion.

## 4. Discussion

As of 11 August 2022, according to the US FDA, there were more than 640 incoming drug development projects, 470 trials in review, 12 treatments approved for emergency use, and 2 approved treatments—including remdesivir and baricitanib. Although lower fatality rates and clinical improvements in the early stages of infection were observed [36,37], these approved drugs showed no significant improvement in response to severe COVID-19 in various patients [38]. The process of SARS-CoV-2 infection begins with the attachment of viral S protein to the human ACE2 receptor, resulting in entry into the cells and causing extensive cell damage. This further triggers cytokine levels, leading to ARDS and multiple organ failure. Considering that prevention is believed to be better than cure, the search for drugs to prevent viral infections and the onset of severe illness requires relentless effort. Learning from the experience from the use of Chinese herbal medicines after decades treating epidemics, there is still an urgent need to provide complementary and alternative treatments for the management of patients with SARS-CoV-2 infection [39,40]. In an effort to rapidly find a drug that can treat COVID-19, we have utilized a bioinformatics approach to rapidly screen for a potential drug, and we identified PG2. Previous studies has revealed transcriptome fingerprints of COVID-19 infections [26], which we can use to screen among our drug response transcriptome library for potential compounds to reverse the disease signature via GSEA. This method can be applied for other diseases to quickly identify potential therapy for future pandemic similar to the COVID-19 situation.

**Figure 7:**
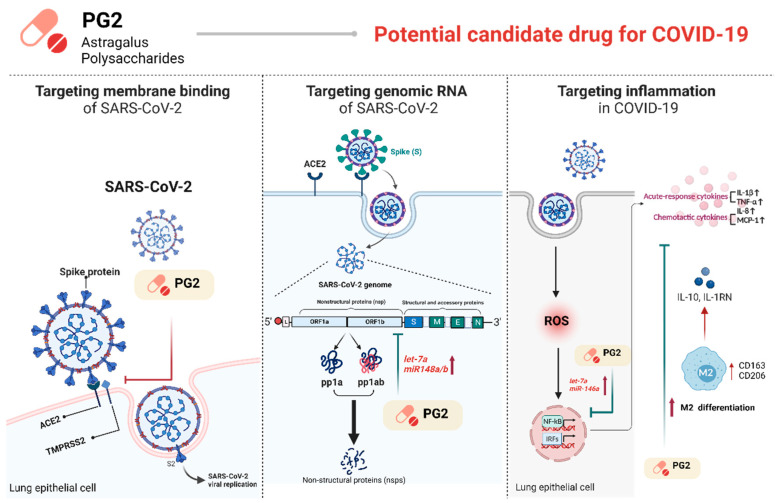
The potential mechanisms of targeting SARS-CoV-2 and ARDS of PG2.

PG2 has been shown in previous clinical trials to be effective in modulating the immune system in humans, as well as having fewer side effects and a high safety profile. To elucidate the potential pharmacological mechanisms and targets of PG2 with suggestions from the GSEA results we discovered that PG2 has the potential to treat COVID-19 by inhibiting its fusion activity, modulating miRNAs in lung epithelial cells, and effectively regulating macrophage activity. A schematic summary of the potential mechanisms of PG2 targeting SARS-CoV-2 and ARDS is shown in Figure 7.

PG2 not only acts as a fusion inhibitor of viral infection in the host cells but also has the potential to inhibit viral replication by inducing *let-7a*, *miR-148b*, and *miR-146a* miRNA expression, stimulating M2 macrophage differentiation and triggering the secretion of anti-inflammatory cytokines, including IL-10 and IL-1RN. Our results are labeled in red, while the potential mechanisms of PG2 are labeled in green. Figures were created using BioRender.com.

Unlike SARS-CoV, which uses the endosomal membrane fusion pathway to infect host cells, SARS-CoV-2-infected cells form the syncytium, suggesting that SARS-CoV-2 can primarily use the plasma membrane fusion pathway to invade and replicate within host cells [3,41]. Consistently, in a cell–cell fusion assay, the SARS-CoV-2 S protein can efficiently regulate syncytium formation between the donor and receiving cells in the absence of exogenous protein hydrolases such as trypsin, whereas the SARS-CoV S protein cannot. In fact, for the majority of viruses, endosomal membrane fusion tends to activate host cell antiviral immunity; therefore, it is less effective than plasma membrane fusion [42,43]. In addition, the S-protein-mediated membrane fusion not only participates in the SARS-CoV-2 entry process but also plays other potential roles in severe COVID-19 pathogenesis, such as promoting viral dissemination, immune escape, and inflammatory response [44]. Therefore, S-protein-mediated membrane fusion is a potent target for the development of anti-SARS-CoV-2 drugs [31]. Recent reports have shown that SARS-CoV-2 primarily uses host proteases, such as TMPRSS2 and furin, for plasma membrane fusion [29,45], and PG2 could effectively inhibit the fusion ability of S protein, which is presumed to be a potential drug for preventing the fusion of viruses and their further progression. The binding of the viral S protein to the human ACE2 receptor initiates the host cellular invasion of SARS-CoV-2, enabled by the receptor-binding domain (RBD) region on S1 of the SARS-CoV-2 S protein during viral binding and the fusion peptide (FP) on S2 responsible for viral fusion [46]. During host cell entry, the linkage between glycans and complexed sugar molecules on the viral surface and host cells via glycoproteins is essential [47]. Although glycans present on the S protein contribute negligibly to viral binding, they play a substantial role in viral cell invasion and fusion with the host cell [48]. Considering that *Astragalus*-based formulas contain polysaccharides, such as β-galactosidase, to explain the role of PG2 in repressing fusion, we hypothesized that PG2 could compete with the S2 subunits or glycans on the surface of S proteins.

Using an ELISA-based binding assay, the interference of trimeric S protein and hACE2 binding in the presence of PG2 was clearly demonstrated. Interestingly, such inhibitory effects were variant-specific, and PG2 appeared to be more effective when confronted with wild-type, alpha, and beta variants (Figure 3). Moreover, the different results of the binding of SARS-CoV-2 S protein ACE2 between cell–cell fusion assay and ELISA binding assay might be due to the assay conditions (Figure 2 and Figure S2). In the cell–cell fusion assay, in addition to ACE2, there are additional cellular factors that interact with SARS-CoV-2 to facilitate or stabilize the binding of the S protein with ACE2 [49]. Furthermore, the SARS-CoV-2-S protein expressed on BHK-21 might undergo post-translational modifications (PTMs), such as glycosylation and palmitoylation, which are critical for SARS-CoV-2-S protein-mediated membrane fusion and infection [50]. Therefore, we speculated that additional host factors and PTMs might reveal not only different results from various experimental approaches but also various responses of SARS-CoV-2 variants to the treatment of PG2.

In addition to inhibiting the fusion ability of S protein, PG2 significantly enhanced miRNA expression in lung epithelial cells. Nearly all biological processes, including development, hemostasis, and inflammation, are regulated by miRNAs. miRNAs are small non-coding RNAs that regulate gene expression by targeting mRNAs [51]. Several miRNAs have been observed to have viral inhibitory effects by targeting the viral RNA genome and/or inhibiting the expression of virus-dependent cellular cofactors [52,53]. PG2 significantly increased the expression levels of *let-7a* in lung epithelial cells (Figure 4A). *Let-7* is a family of human cells consisting of 13 members. *Let-7a* has been reported to suppress IL-6 expression [54,55], an abundant inflammatory factor induced by SARS-CoV-2. Furthermore, it has also been reported that in THP-1 cells *pri-let-7a* overexpressing, *let-7a* not only decreased the *IL-6* mRNA level but also significantly reduced the expression of several other SARS-CoV-2 related pro-inflammatory cytokines and chemokines, including IL-1β, IL-8, GM-CSF, and TNF-α [56]. In addition to cytokine storm inhibition, *let-7a* was also believed to be effective in inhibiting SARS-CoV-2 RNA and protein expression, as well as viral replication [57]. Our results also demonstrated that PG2 could be a potential drug to inhibit the replication of SARS-CoV-2 in lung epithelial cells and suppress cytokine storms by inducing the expression of *let-7a*.

Additionally, we used the Mienturent database, an interactive web tool for miRNA-target enrichment and network-based analysis, to identify the signaling pathways regulated by *hsa-let-7a-5p*, *hsa-miR-148b-5p*, and *hsa-miR-146a-5p*. The genes targeted by these miRNAs were enriched to the AP-1 transcription factor network and the forkhead box O (FoxO) signaling pathway (Supplement Table S1). A previous study showed that the SARS-CoV accessory protein induces AP-1 transcriptional activity through the activation of the c-Jun N-terminal kinase (JNK) pathway [58]. Inhibition of NF-κB/JNK would be effective against sepsis-induced acute lung injury [59]. Currently, novel agents that suppress NF-κB/JNK signaling, including tocilizumab and baricitinib, have been approved for the treatment of SARS-CoV-2 [60,61]. Therefore, the direct effects of PG2 on these inflammatory pathways were investigated.

Macrophages are ubiquitous in human organs and are present in large numbers in the lungs. Monocytes of bone marrow origin differentiate into alveolar macrophages, which serve as the first line of defense against invading organisms, such as pathogens and harmful substances [62,63,64]. Macrophages can be divided into two functional phenotypes. Excessive pathogenic infections may lead to enhanced M1 macrophage activity, resulting in the accelerated and massive production of inflammatory cytokines and chemokines, further leading to cytokine storms. In contrast, the activation of M2 macrophages triggers the release of anti-inflammatory cytokines, thereby limiting inflammation and promoting tissue repair [65]. Persistent enrichment of the alveolar space with pro-inflammatory monocytes and continued drive of alveolar inflammation has been observed in some patients infected with SARS-CoV-2 who developed severe pneumonia and ARDS [66]. To determine whether PG2 could effectively promote the conversion of macrophages to an anti-inflammatory phenotype, we differentiated THP-1 cells into THP-1 macrophages and observed that PG2 could effectively enhance the expression of CD163 (a marker of M2 macrophages) in THP-1 macrophages, indicating M2 polarization of PG2, which highlighted the anti-inflammatory effect of PG2. The clinical efficacy of anti-IL-6R in COVID-19 patients has been tested in a recent randomized, double-blind phase III COVACTA trial (NCT04320615; clinicaltrials.gov) but showed an insignificant decrease in fatality [67]. This is further evidence that cytokines other than IL-6 are involved in regulating pathological mechanisms. Considering the broad anti-inflammatory effects of M2 macrophages, M2-polarizing PG2 may play a vital role in balancing the inflammatory environment. It is alarming that convalescent patients, even those with mild COVID-19, can sustain persistent symptoms for months post-infection, referred to as “long COVID” [68]. No approved treatments are available for these patients. However, anti-inflammatory treatment is a potential medical support that can be provided to these patients, which may support our hypothesis that PG2 is a candidate for treating long-term COVID cases [69].

Accumulating data have revealed that cytokine storm is often related to COVID-19 pneumonia and its exacerbation in severe cases [70]. In particular, the innate immune response, which is mostly regulated by macrophages in response to SARS-CoV-2 infection, possibly contributes to ARDS [71]. Depending on their microenvironment, they are polarized to either the classically activated phenotype (M1) or alternatively activated phenotype (M2). It has been previously established that during SARS-CoV-2 infection, M2 macrophages are essential innate cells. Suppressing viral invasion by an anti-ACE2 antibody robustly improved the efficiency of M2 macrophages [6].

M2 macrophages are often polarized by IL-4 and/or IL-13 and exhibit high surface expression of CD163 and CD206. Our culture system revealed that PG2-induced human monocytes polarize into M2 macrophages and produce anti-inflammatory cytokines in the absence of IL-4 and/or IL-13 stimulation. In contrast, PG2-induced M2 macrophages exhibited similar phenotypes, as the levels of anti-inflammatory cytokines IL-10 and IL-1RN were substantially induced. These similar immune response patterns of M2 macrophages may suggest that PG2 can activate M2 macrophage differentiation but is independent of IL-4Ra and/or IL-13Ra signaling. Finally, PG2 can stimulate M2 macrophage activation and exert anti-inflammatory properties to prevent the pathogenesis of cytokine storms in lung damage caused by COVID-19.

In conclusion, our bioinformatics platform delineates the moonlight role of PG2 via reversing the SARS-CoV-2 infection signatures. Empirically, PG2 not only blocks the binding of WT, alpha, and beta S proteins to recombinant ACE2 while suppressing WT viral entry into the host cells but also promotes M2 macrophage polarization and anti-inflammatory cytokine production. Our study rationalizes the efficacy of PG2 treatment in patients with severe COVID-19 symptoms due to a decrease in NLR. Considered together, these results suggest that PG2 is a potent repurposed drug with multiple effects in SARS-CoV-2-infected patients in the absence of effective approved drugs for COVID-19 treatment.

## Figures and Tables

**Table 1:** GSEA analysis of the PG2 treatment transcriptional profile reveals PG2 as the potential option for COVID-19 patients.

Gene Set Name	NES *	*p*-Val	FDR q-Val
Genes upregulated in five primary endothelial cell types (lung, aortic, iliac, dermal, and colon) by TNF	−3.06	0.00	0.00
Genes strongly upregulated in postmortem lung tissue from COVID-19 patients vs. uninfected biopsy.	−2.58	0.00	0.00
Genes upregulated in ARDS	−2.32	0.00	0.00
Genes upregulated on infection of normal human bronchial epithelial cells by SARS-CoV-2	−2.23	0.00	0.00
Genes upregulated in SARS-CoV-2 infection in Calu-3 cells	−1.98	0.00	0.01
Type I Interferon Induction and Signaling During SARS-CoV-2 Infection	−1.56	0.02	0.07
Ferroptosis	−1.70	0.01	0.04
Iron uptake and transport	−1.60	0.01	0.06
COVID-19 Adverse Outcome Pathway	−1.45	0.08	0.12
Translation of SARS-CoV-2 structural proteins	−1.36	0.08	0.16
Maturation or SARS-CoV-2 spike protein	−1.35	0.11	0.16
SARS-CoV-2 Infection	−1.27	0.09	0.22

* NES: Normalized enrichment score.

## Data Availability

The microarray data are available in the Supplementary File.

## Supplementary Materials

The following supporting information can be downloaded at: https://www.mdpi.com/article/10.3390/v15030641/s1, Figure S1: GSEA reveals anti-SARS-CoV-2 infection effect by PG2; Figure S2: The expression of ACE2 and TMPRSS2 in Calu-3 with PG2 treatment; Figure S3: Cell viability of BEAS-2B, BHK-21, and Calu-3 cells after PG2 treatment; Table S1: *Hsa-let-7a-5p*, *hsa-miR-148b-5p*, *hsa-miR-146a-5p* were enriched to AP-1 transcription factor network and FoxO signaling pathway.
