# Supplementary material for: Repurposing Astragalus Polysaccharide PG2 for Inhibiting ACE2 and SARS-CoV-2 Spike Syncytial Formation and Anti-Inflammatory Effects
Source: Viruses. 2023 Feb 27;15(3):641. doi: 10.3390/v15030641 (PMC10054482; doi:10.3390/v15030641)
Supplement: Supplementary file 1 [file viruses-15-00641-s001.zip › Supplementary_Material_v4.pdf]

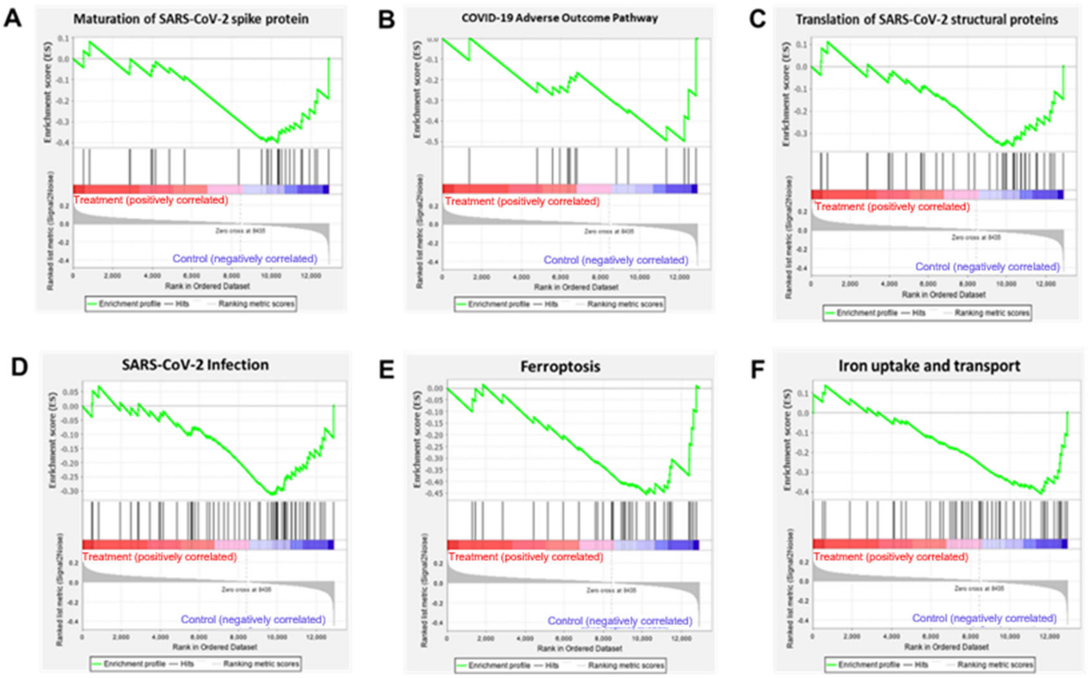


**Supplementary Figure S1. GSEA reveals anti-SARS-CoV-2 infection effect by PG2.** PG2 treatment GSEA enrichment plot of gene set regarding (**A**) Maturation of SARS-CoV-2 spike protein (Reactome - R-HSA-9694548); (**B**) COVID-19 adverse outcome pathway (WikiPathways - WP4891); (**C**) translation of SARS-CoV-2 structural proteins (Reactome - R-HSA-9694635); (**D**) SARS-CoV-2 Infection (Reactome - R-HSA-9694516); (**E**) Ferroptosis (WikiPathways - WP4313); and (**F**) Iron uptake and transport (Reactome - R-HSA-917937).

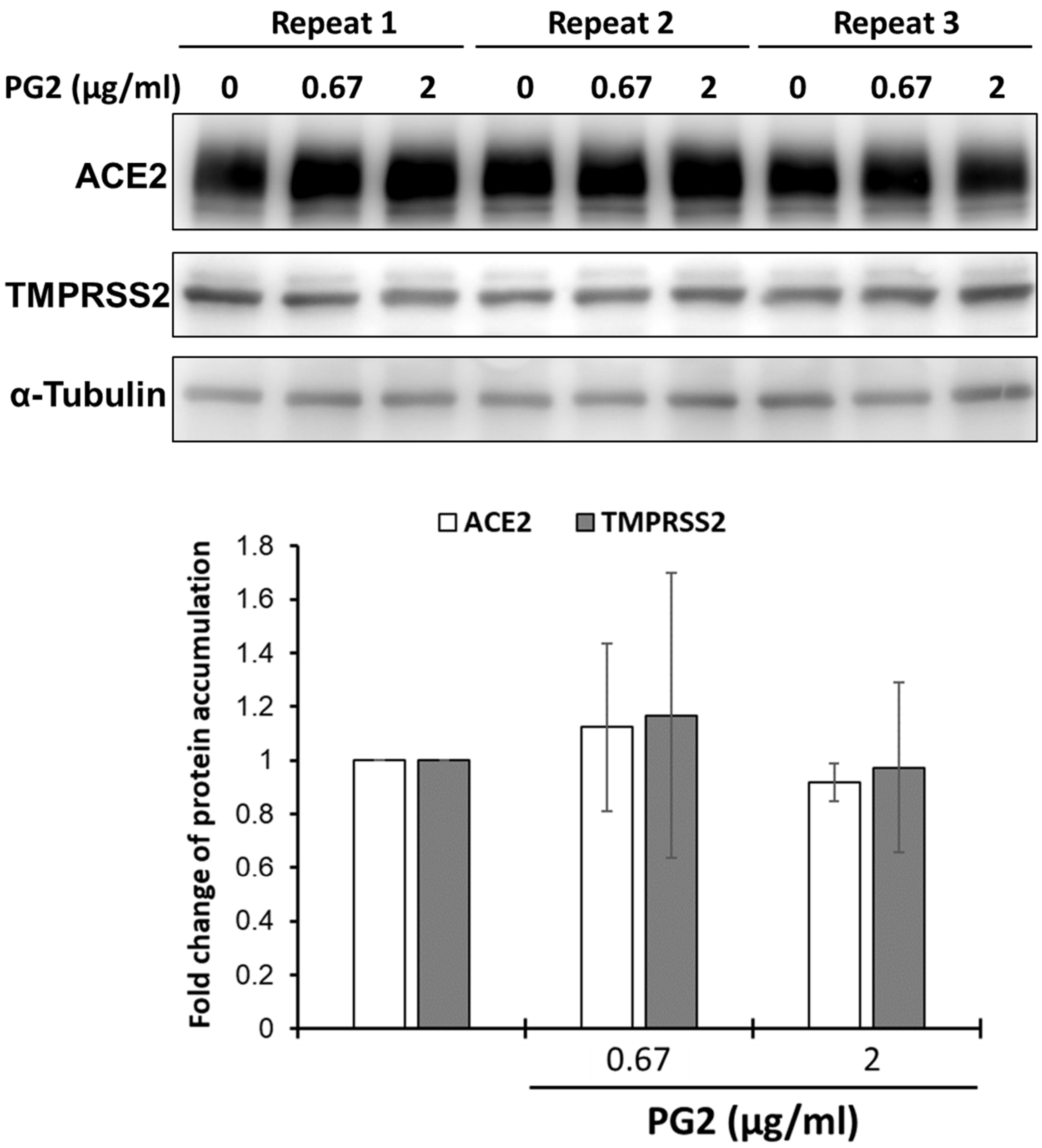


**Supplementary Figure S2. The expression of ACE2 and TMPRSS2 in Calu-3 with PG2 treatment.** (**A**) Calu-3 cells incubated with 0, 0.67, and 2 mg/ml PG2, respectively, for 5 h. The amount of ACE2 and TMPRSS2 in Calu-3 were detected using western blotting and normalized by $\alpha$-Tubulin expression. (**B**) The histogram depicts the average fold change of ACE2 and TMPRSS2 expression in each group compared with the untreated group (0 mg/ml).

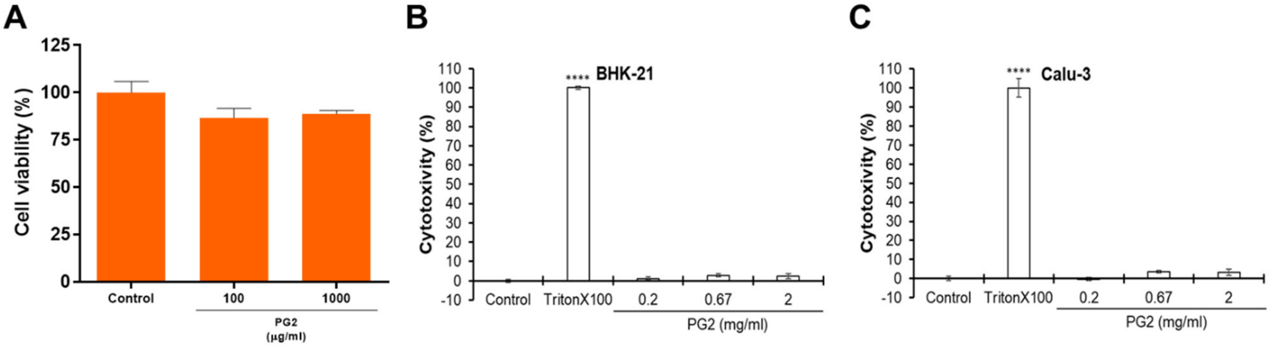


**Supplementary Figure S3. Cell viability of BEAS-2B, BHK-21, and Calu-3 cells after PG2 treatment.** Cell viability of BEAS-2B cells after 24 h of PG2 treatment by SRB assay (A), and BHK-21 (B) and Calu-3 (C) cells after 5 h of PG2 treatment by LDH cytotoxicity assay. *$p$ <0.05, **$p$ <0.01, and ***$p$ <0.001.

**Supplementary Table S1.** ***Has-let-7a-5p*, *hsa-miR-148b-5p*, *hsa-miR-146a-5p* were enriched to AP-1 transcription factor network and FoxO signaling pathway.**

| **Step 1. submit miRNA** | **Step 2. Mienturnet Enrichment results miRTarBase** | | | | | | **Step 3. the enrichment from CPDB database** | | | | |
|---|---|---|---|---|---|---|---|---|---|---|---|
| hsa-let-7a-5p | Mienturnet Enrichment results miRTarBase | | | | | | pathway | p-value | q-value | source | members_input_overlap |
| hsa-miR-148b-5p | **Gene Symbol** | **p-value** | **FDR** | **Odd ratio** | **microRNA 1** | **microRNA 2** | Photodynamic therapy-induced AP-1 survival signaling. | 1.34E-08 | 2.14E-06 | Wikipathways | CCND1; IL6; CDKN1A; BCL2L11; EGFR |
| hsa-miR-146a-5p | GPM6B | 0.0001 | 0.026 | 0.009234321 | hsa-let-7a-5p | hsa-miR-146a-5p | DNA Damage Response (only ATM dependent) | 1.57E-08 | 2.14E-06 | Wikipathways | PMAIP1; CCND1; CDKN1A; CCND2; NFKB1; BCL2L11 |
| | NFKB1 | 0.0001 | 0.026 | 0.009811466 | hsa-miR-146a-5p | hsa-let-7a-5p | Hepatitis C and Hepatocellular Carcinoma | 2.39E-08 | 2.18E-06 | Wikipathways | CCND1; CXCL8; CDKN1A; IL6; NFKB1 |
| | UHRF1 | 8E-05 | 0.026 | 0.008080031 | hsa-miR-146a-5p | hsa-let-7a-5p | FoxO signaling pathway - Homo sapiens (human) | 4.49E-08 | 2.83E-06 | KEGG | BCL2L11; CCND1; CDKN1A; CCND2; EGFR; IL6 |
| | ZNF629 | 0.0001 | 0.026 | 0.009811466 | hsa-let-7a-5p | hsa-miR-146a-5p | Pathways in cancer - Homo sapiens (human) | 5.19E-08 | 2.83E-06 | KEGG | PMAIP1; CCND1; CXCL8; CDKN1A; CCND2; EGFR; IL6; NFKB1; BCL2L11 |
| | COX2 | 0.0002 | 0.0291 | 0.011542901 | hsa-let-7a-5p | hsa-miR-146a-5p | AP-1 transcription factor network | 7.47E-08 | 3.40E-06 | PID | CCND1; CXCL8; BCL2L11; DUSP1; IL6 |
| | IL6 | 0.0002 | 0.0294 | 0.012697191 | hsa-let-7a-5p | hsa-miR-146a-5p | Cellular senescence - Homo sapiens (human) | 1.48E-07 | 5.77E-06 | KEGG | CCND1; CXCL8; CDKN1A; CCND2; IL6; NFKB1 |
| | AKAP8 | 0.0005 | 0.0322 | 0.020200077 | hsa-let-7a-5p | hsa-miR-146a-5p | Colorectal cancer - Homo sapiens (human) | 2.11E-07 | 7.22E-06 | KEGG | CCND1; BCL2L11; CDKN1A; PMAIP1; EGFR |
| | C1orf21 | 0.0007 | 0.0322 | 0.023085802 | hsa-miR-146a-5p | hsa-let-7a-5p | Photodynamic therapy-induced NF-kB survival signaling | 2.56E-07 | 7.75E-06 | Wikipathways | CCND1; CXCL8; IL6; NFKB1 |
| | CXCL8 | 0.0006 | 0.0322 | 0.020777222 | hsa-miR-146a-5p | hsa-let-7a-5p | Bladder Cancer | 4.44E-07 | 1.21E-05 | Wikipathways | CCND1; CXCL8; CDKN1A; EGFR |
| | DUSP1 | 0.0004 | 0.0322 | 0.017314352 | hsa-miR-146a-5p | hsa-let-7a-5p | Bladder cancer - Homo sapiens (human) | 4.91E-07 | 1.22E-05 | KEGG | CCND1; CXCL8; CDKN1A; EGFR |
| | EGFR | 0.0007 | 0.0322 | 0.023085802 | hsa-miR-146a-5p | hsa-let-7a-5p | Epstein-Barr virus infection - Homo sapiens (human) | 5.52E-07 | 1.26E-05 | KEGG | BCL2L11; CCND1; CDKN1A; CCND2; IL6; NFKB1 |
| | GPRIN2 | 0.0004 | 0.0322 | 0.018468642 | hsa-miR-146a-5p | hsa-miR-148b-5p | PI3K-Akt Signaling Pathway | 6.57E-07 | 1.38E-05 | Wikipathways | BCL2L11; CCND1; CDKN1A; CCND2; EGFR; IL6; NFKB1 |
| | LIMD2 | 0.0005 | 0.0322 | 0.020200077 | hsa-miR-146a-5p | hsa-let-7a-5p | PI3K-Akt signaling pathway - Homo sapiens (human) | 8.62E-07 | 1.68E-05 | KEGG | BCL2L11; CCND1; CDKN1A; CCND2; EGFR; IL6; NFKB1 |
| | ZNF260 | 0.0008 | 0.0322 | 0.025394382 | hsa-let-7a-5p | hsa-miR-146a-5p | Transcriptional regulation by RUNX3 | 1.03E-06 | 1.87E-05 | Wikipathways | CCND1; CDKN1A; BCL2L11 |
| | ZNF738 | 0.0005 | 0.0322 | 0.019045787 | hsa-let-7a-5p | hsa-miR-146a-5p | Human cytomegalovirus infection - Homo sapiens (human) | 1.10E-06 | 1.88E-05 | KEGG | CCND1; CXCL8; CDKN1A; EGFR; IL6; NFKB1 |
| | AREL1 | 0.0015 | 0.0384 | 0.034051558 | hsa-miR-148b-5p | hsa-let-7a-5p | ATF-2 transcription factor network | 2.32E-06 | 3.72E-05 | PID | IL6; CXCL8; CCND1; DUSP1 |
| | PTGES2 | 0.0017 | 0.0384 | 0.035782993 | hsa-let-7a-5p | hsa-miR-146a-5p | Hepatitis B - Homo sapiens (human) | 2.75E-06 | 4.17E-05 | KEGG | CCND1; IL6; CDKN1A; CXCL8; NFKB1 |
| | NSD1 | 0.0025 | 0.0416 | 0.044440169 | hsa-let-7a-5p | hsa-miR-148b-5p | LTF danger signal response pathway | 3.47E-06 | 4.98E-05 | Wikipathways | IL6; CXCL8; NFKB1 |
| | EDEM3 | 0.0027 | 0.0422 | 0.045594459 | hsa-let-7a-5p | hsa-miR-146a-5p | DNA Damage Response | 3.84E-06 | 5.24E-05 | Wikipathways | CCND1; CDKN1A; CCND2; PMAIP1 |
| | ATG9A | 0.0038 | 0.0456 | 0.054251635 | hsa-let-7a-5p | hsa-miR-146a-5p | p53 signaling pathway - Homo sapiens (human) | 4.83E-06 | 6.01E-05 | KEGG | CCND1; CDKN1A; CCND2; PMAIP1 |
| | BCL2L11 | 0.0064 | 0.0534 | 0.070411697 | hsa-let-7a-5p | hsa-miR-148b-5p | JAK-STAT signaling pathway - Homo sapiens (human) | 4.90E-06 | 6.01E-05 | KEGG | CCND1; IL6; CDKN1A; CCND2; EGFR |
| | KDM6B | 0.0073 | 0.0552 | 0.075606002 | hsa-let-7a-5p | hsa-miR-146a-5p | Chromosomal and microsatellite instability in colorectal cancer | 5.11E-06 | 6.01E-05 | Wikipathways | CCND1; BCL2L11; CDKN1A; PMAIP1 |
| | PEG10 | 0.0081 | 0.0552 | 0.079646018 | hsa-miR-148b-5p | hsa-let-7a-5p | Vitamin D in inflammatory diseases | 5.49E-06 | 6.01E-05 | Wikipathways | IL6; NFKB1; DUSP1 |
| | PMAIP1 | 0.0092 | 0.0557 | 0.084840323 | hsa-miR-146a-5p | hsa-let-7a-5p | Pancreatic cancer - Homo sapiens (human) | 5.69E-06 | 6.01E-05 | KEGG | CCND1; NFKB1; CDKN1A; EGFR |
| | CCND2 | 0.0135 | 0.0652 | 0.103308965 | hsa-let-7a-5p | hsa-miR-146a-5p | MicroRNAs in cancer - Homo sapiens (human) | 5.73E-06 | 6.01E-05 | KEGG | BCL2L11; CCND1; CDKN1A; CCND2; EGFR; NFKB1 |
| | ZNF264 | 0.023 | 0.0776 | 0.135629088 | hsa-let-7a-5p | hsa-miR-148b-5p | cyclins and cell cycle regulation | 6.31E-06 | 6.38E-05 | BioCarta | CCND1; CDKN1A; CCND2 |
| | CCND1 | 0.0261 | 0.0804 | 0.144863409 | hsa-let-7a-5p | hsa-miR-146a-5p | Glucocorticoid receptor regulatory network | 7.36E-06 | 7.18E-05 | PID | IL6; NFKB1; CDKN1A; CXCL8 |
| | CDKN1A | 0.0444 | 0.0884 | 0.191035013 | hsa-miR-146a-5p | hsa-let-7a-5p | Signaling Pathways in Glioblastoma | 8.53E-06 | 8.03E-05 | Wikipathways | CCND1; CDKN1A; CCND2; EGFR |
